# Incidence of blast phase in myelofibrosis according to anemia severity

**DOI:** 10.1002/jha2.745

**Published:** 2023-07-17

**Authors:** Barbara Mora, Margherita Maffioli, Elisa Rumi, Paola Guglielmelli, Marianna Caramella, Andrew Kuykendall, Francesca Palandri, Alessandra Iurlo, Valerio De Stefano, Jean‐Jacques Kiladjian, Elena M. Elli, Nicola Polverelli, Jason Gotlib, Francesco Albano, Richard T. Silver, Giulia Benevolo, David M. Ross, Timothy Devos, Oscar Borsani, Tiziano Barbui, Matteo G. Della Porta, Lorenza Bertù, Rami Komrokji, Alessandro M. Vannucchi, Francesco Passamonti

**Affiliations:** ^1^ Department of Oncology, ASST Sette Laghi Ospedale di Circolo Varese Italy; ^2^ Department of Molecular Medicine University of Pavia Pavia Italy; ^3^ Hematology Fondazione IRCCS Policlinico San Matteo Pavia Italy; ^4^ Center of Research and Innovation of Myeloproliferative Neoplasms University of Florence Florence Italy; ^5^ Hematology ASST Grande Ospedale Metropolitano Niguarda Milan Italy; ^6^ Malignant Hematology Department, Blood and Marrow Transplantation H. Lee Moffitt Cancer Center and Research Institute Tampa Florida USA; ^7^ Institute of Hematology “Seràgnoli” IRCCS Azienda Ospedaliero‐Universitaria di Bologna Bologna Italy; ^8^ Hematology Foundation IRCCS Ca'Granda Ospedale Maggiore Policlinico Milan Italy; ^9^ Hematology Fondazione Policlinico Universitario A. Gemelli IRCCS Rome Italy; ^10^ Hôpital Saint‐Louis Université Paris Cité Paris France; ^11^ Division of Hematology and Bone Marrow Unit IRCCS San Gerardo dei Tintori Monza Italy; ^12^ Unit of Blood Diseases and Stem Cell Transplantation ASST Spedali Civili di Brescia Brescia Italy; ^13^ Division of Hematology, Stanford Cancer Institute Stanford University School of Medicine Stanford California USA; ^14^ Hematology ‐ Department of Emergency and Organ Transplantation University of Bari Bari Italy; ^15^ Richard T. Silver Myeloproliferative Neoplasms Center NewYork‐Presbyterian Weill Cornell Medical Center New York New York USA; ^16^ Hematology Unit AOU Città della Salute e della Scienza di Torino Turin Italy; ^17^ Haematology Directorate, SA Pathology Royal Adelaide Hospital Adelaide South Australia Australia; ^18^ Department of Hematology KU Leuven University Hospitals Leuven Leuven Belgium; ^19^ FROM Research Foundation ASST Papa Giovanni XXIII Bergamo Italy; ^20^ Department of Biomedical Sciences Humanitas University Pieve Emanuele Italy; ^21^ Department of Medicine and Surgery University of Insubria Varese Italy; ^22^ Department of Oncology and Haemato‐Oncology University of Milan Milan Italy

**Keywords:** acute myeloid leukemia, essential thrombocythemia, myelofibrosis, polycythemia vera, ruxolitinib

## Abstract

Myelofibrosis (MF) is a clonal malignancy frequently characterized by anemia and in 10%–20% of cases it can evolve into blast phase (BP). Anemia in MF is associated with reduced survival and ‐in primary MF‐ also with an increased probability of BP. Conventional treatments for anemia have limited effectiveness in MF.

Within a dataset of 1752 MF subjects largely unexposed to ruxolitinib (RUX), BP incidence was 2.5% patients per year (p‐y). This rate reached respectively 4.3% and 4.5% p‐y in case of patients with common terminology criteria for adverse events (CTCAE) grade 3/4 and grade 2 anemia, respectively, that represented together 32% of the cohort. Among 273 MF cases treated with RUX, BP incidence was 2.89% p‐y and it reached 4.86% p‐y in subjects who started RUX with CTCAE grade 2 anemia (one third of total). Within patients with red blood cell transfusion‐dependency at 6 months of RUX (21% of the exposed), BP rate was 4.2% p‐y. Our study highlights a relevant incidence of BP in anemic MF patients, with a similar rate whether treated with or without RUX. These findings will help treating physicians to make decisions on the safety profile of innovative anemia treatments.

## 1 INTRODUCTION

Myelofibrosis (MF) is a Philadelphia‐negative myeloproliferative neoplasm (MPN) characterized by a variety of blood cell alterations, splenomegaly, constitutional symptoms, bone marrow fibrosis (BMF), and a tendency to develop blast phase (BP) [1, 2, 3]. MF comprises primary MF (PMF), categorized as prefibrotic‐ (pre) or overt‐PMF, and secondary MF (SMF), that encompasses post‐polycythemia vera (PPV‐), and postessential thrombocythemia (PET‐) MF [1]. Median survival of the pre‐PMF, overt‐PMF, and SMF subtypes is around 14 years, seven and 9 years, respectively [4, 5, 6, 7]. The main causes of mortality are nonclonal disease progression and evolution into BP [4, 5, 6, 7]. The latter occurs in 10%–20% of MF patients [8, 9], with an incidence that has not changed in recent times [8, 10, 11].

Anemia is a characteristic feature of MF, found in around 35%–40% of cases at diagnosis, becoming more frequent with the disease progression, and impacting deeply on patients’ quality of life [12, 13, 14, 15]. In addition, by using the currently approved JAK inhibitors (JAKis), such as ruxolitinib (RUX) or fedratinib (FED), hemoglobin (Hb) reduction is an expected early on‐target effect, with grade 3/4 anemia occurring in 38–45% of cases [2]. Anemia is listed among the major survival risk factors in MF prognostic models developed before the widespread use of JAKis [5, 7, 12]. In this respect, red blood cell (RBC) transfusion requirement represented a detrimental factor for survival in 209 RUX‐treated MF patients [16]. In large series of PMF patients, risk factors for BP evolution have been investigated and, among those, anemia has been recognized as relevant [9, 17, 18]. On this point, information in SMF or in RUX‐treated cases is more limited [6, 11].

In current practice, treatment of MF‐associated anemia mostly consists of erythropoiesis‐stimulating agents, danazol and RBC transfusions [2, 19]. However, the interest of studying anemia‐improving molecules with new targets is growing in MF, although the impact of such treatments on BP occurrence is unknown [2, 19].

In this study, we reported the incidence of BP according to anemia severity in large real‐world cohorts of PMF and SMF patients, treated with or without JAKis, mainly RUX. This could serve as a reference for assessing BP occurrence in populations of MF patients receiving innovative anemia‐oriented treatments.

## 2 MATERIALS AND METHODS

### 2.1 Study populations and definitions

A total of 2381 MF patients entered the analysis, and we generated two different cohorts (Table S1). Cohort 1 (C1) was composed of (1) 331 PMF patients from the Institutional MPN database of the Hematology Unit, University of Insubria, Varese, Italy (MPN‐VA dataset); (2) 519 overt‐PMF cases that built the Dynamic International Prognostic Scoring System (DIPSS) model [12]; (3) 1258 SMF subjects from the MF Secondary to polycythemia vera and essential thrombocythemia (MYSEC) project [7, 20]. C1 also included patients who received JAKis after 6 months from MF diagnosis. Cohort 2 (C2) included 273 RUX‐treated PMF and SMF patients from the Italian real‐world ambispective observational RUXOREL‐MF study [16]. Detailed information on the DIPSS, MYSEC and RUXOREL‐MF populations are reported in related papers [12, 16, 20]. Then, we selected 2025 patients (1752 for C1 (Table 1) and 273 for C2 (Table 2)) who had demographic, clinical and hematologic data collected either at diagnosis (C1) or at time of RUX start (C2), with at least 6 months of follow‐up and with updated information on stem cell transplantation (SCT), BP evolution and death.

**TABLE 1:** Main features at diagnosis and follow‐up events of 1752 myelofibrosis patients in Cohort 1 overall and stratified by anemia degree according to the CTCAE classification.

			CTCAE Hb classification			
		Total	Grade 3/4 anemia	Grade 2 anemia	Grade 1/no anemia	*p*‐Value
Patients	*n* (%)	1752 (100)	131 (7.5)	427 (24.4)	1194 (68.1)	
Pre‐PMF	*n* (%)	136 (7.8)	1 (0.8)	7 (1.6)	128 (10.7)	<0.0001
Overt‐PMF	*n* (%)	638 (36.4)	74 (56.5)	160 (37.5)	404 (33.8)	
PET‐MF	*n* (%)	531 (30.3)	41 (31.3)	170 (39.8)	320 (26.8)	
PPV‐MF	*n* (%)	447 (25.5)	15 (11.5)	90 (21.1)	342 (28.6)	
Age at diagnosis (years)	Mean (SD)	62.4 (12.8)	64.7 (10.6)	65.9 (11.8)	60.9 (13.1)	<0.0001
	Median (IQR)	64 (55–72)	66 (57–72)	67 (58–74)	63 (53–71)	
Male gender	*n* (%)	987 (56.3)	98 (74.8)	219 (51.3)	679 (56.1)	<0.0001
Palpable spleen	*n* (%)	1292 (77.1)	100 (80.0)	316 (79.0)	876 (76.1)	0.36
Palpable spleen (cm)	Mean (SD)	7.8 (5.4)	8.1 (5.7)	7.6 (5.5)	7.9 (5.4)	0.58
	Median (IQR)	6 (4–11)	7 (3–10)	6 (3–11)	7 (4–11)	
Constitutional symptoms	*n* (%)	532 (30.4)	77 (58.8)	153 (35.8)	302 (25.3)	<0.0001
Hb (g/dL)	Mean (SD)	11.3 (2.4)	6.9 (0.9)	9.1 (0.6)	12.6 (1.6)	<0.0001
	Median (IQR)	11.4 (9.6–13.1)	7.2 (6.3–7.6)	9.2 (8.7–9.7)	12.4 (11.3–13.8)	
Circulating blasts (%)	Mean (SD)	0.8 (2.1)	1.4 (2.8)	1.0 (2.4)	0.6 (1.9)	<0.0001
	Median (IQR)	0.0 (0.0–0.1)	0 (0–2)	0 (0–1)	0 (0–0)	
WBC (x10^9/L)	Mean (SD)	13.0 (11.3)	8.7 (10.0)	12.3 (12.3)	13.7 (11.0)	<0.0001
	Median (IQR)	9.6 (6.2–15.5)	5.7 (3.3–9.5)	8.0 (5.0–15.0)	10.5 (7.1–16.4)	
PLT count (x10^9/L)	Mean (SD)	415.6 (316.9)	247.9 (243.2)	347.4 (287.3)	457.4 (324.0)	<0.0001
	Median (IQR)	349 (183–565.5)	160 (72–336)	253 (143–485)	395 (221–603)	
PLT count < 100 (x10^9/L)	*n* (%)	190 (10.9)	44 (33.6)	68 (16.1)	78 (6.6)	<0.0001
Low‐intermediate 1 risk	*n* (%)	1140 (66.4)	28 (22.2)	128 (31.2)	984 (83.4)	<0.0001
Intermediate 2‐ high risk	*n* (%)	576 (33.6)	98 (77.8)	282 (68.8)	196 (16.6)	
JAKi exposure during time	*n* (%)	285 (16.3)	19 (14.5)	54 (12.7)	212 (17.8)	0.04
Time to JAKi start (years)	Mean (SD)	3.9 (3.8)	2.2 (1.7)	3.4 (3.4)	4.1 (3.9)	0.04
	Median (IQR)	2.7 (1.2–5.0)	1.4 (1.1–2.6)	2.2 (0.9–4.6)	2.9 (1.3–5.5)	
SCT	*n* (%)	87 (5.0)	12 (9.2)	24 (5.6)	51 (4.3)	0.04
Follow‐up (year)	Median (IQR)	4.0 (1.9–7.2)	2.4 (1.2–5.1)	2.9 (1.2–5.6)	4.7 (2.2–8.2)	<0.0001
Deaths	*n* (%)	890 (50.8)	103 (78.6)	271 (63.5)	516 (43.2)	<0.0001
Time to BP (years)	Median (IQR)	2.2 (0.9–3.9)	1.7 (0.8–3.9)	1.7 (0.6–3.8)	2.6 (1.0–4.2)	0.16

*Note*: Percentage calculated on number of patients with data available for each variable.

Abbreviations: BP, blast phase; CTCAE, common terminology criteria for adverse events; Hb, hemoglobin; IQR, interquartile range; JAKi, JAK inhibitors; MF, myelofibrosis; n, number; overt‐PMF, overt‐primary myelofibrosis; PET‐MF, post‐essential thrombocythemia myelofibrosis; PLT, platelets; PPV‐MF, post‐polycythemia vera myelofibrosis; pre‐PMF, prefibrotic‐primary myelofibrosis; SCT, stem cell transplant; SD, standard deviation; WBC, white blood cells count.

**TABLE 2:** Main features at ruxolitinib start and follow‐up events of 273 patients in Cohort 2, overall and stratified by red blood cells‐transfusion dependency and by anemia degree according to the CTCAE classification.

			RBC‐TD status			CTCAE Hb classification			
		Total	TD	no‐TD	*p*‐Value	<8 g/dL	8‐10 g/dL	>10 g/dL	*p*‐Value
Patients	*n* (%)	273 (100)	41 (15)	232 (85)		21 (7.7)	88 (32.2)	164 (60.1)	
Age at diagnosis (years)	Mean (SD)	60.9 (12.1)	63.3 (11.4)	60.4 (12.2)	0.13	63.3 (10.4)	61.1 (11.2)	60.5 (10.9)	0.48
	Median (IQR)	62 (55–69)	65 (58–71)	62 (54–69)		66 (58–71)	63 (55–68)	62 (54–69)	
Male gender	*n* (%)	168 (61.5)	29 (70.7)	139 (59.9)	0.19	15 (71.4)	59 (67.1)	94 (57.3)	0.20
Palpable spleen	*n* (%)	259 (97.7)	40 (100.0)	219 (97.3)	0.60	21 (100.0)	83 (98.8)	155 (96.9)	0.80
Palpable spleen (cm)	Mean (SD)	11.2 (5.6)	12.4 (6.1)	11.0 (5.5)	0.24	11.0 (4.6)	11.8 (6.0)	10.9 (5.4)	0.78
	Median (IQR)	11 (7–15)	12 (8–15.5)	10 (7–14)		10 (8–12)	11 (7–16)	11 (7–14.5)	
Constitutional symptoms	*n* (%)	177 (64.8)	30 (73.2)	147 (63.4)	0.22	14 (66.7)	53 (60.2)	110 (67.1)	0.55
Hb (g/dL)	Mean (SD)	10.7 (2.0)	8.2 (1.1)	11.2 (1.8)	<0.0001	7.1 (0.5)	9.2 (0.6)	12.0 (1.4)	<0.0001
	Median (IQR)	10.5 (9.3–12.3)	8.4 (7.3–8.9)	11 (9.9–12.6)		7.2 (6.9–7.5)	9.2 (8.8–9.7)	11.6 (10.9–13.2)	
Hb > 10 (g/dL)	*n* (%)	164 (60.1)	2 (4.9)	162 (69.8)	<0.0001				
Hb 8–10 (g/dL)	*n* (%)	88 (32.2)	23 (56.1)	65 (28.0)					
Hb < 8 (g/dL)	*n* (%)	21 (7.7)	16 (39.0)	5 (2.2)					
Mild/no anemia	*n* (%)	141 (51.7)	1 (2.4)	140 (60.3)	<0.0001				
Moderate anemia	*n* (%)	91 (33.3)	13 (31.7)	78 (33.6)					
Severe anemia	*n* (%)	41 (15.0)	27 (65.9)	14 (6.0)					
Hb ≤9.5 (g/dL)	*n* (%)	79 (28.9)	37 (90.2)	42 (18.1)	<0.0001				
WBC (x10^9/L)	Mean (SD)	14.3 (11.2)	10.6 (6.8)	15.0 (11.7)	0.03	9.8 (7.0)	14.9 (13.5)	14.5 (10.2)	0.02
	Median (IQR)	11 (6.6–17.4)	9.9 (5.1–14.5)	11 (7.2–18.5)		7.2 (4.1–13.7)	9.7 (5.8–17.9)	11.7 (7.5–27.2)	
Circulating blasts (%)	Mean (SD)	1.5 (2.2)	2.2 (2.8)	1.4 (2.1)	0.06	2.3 (3.3)	1.8 (2.3)	1.3 (2.0)	0.11
	Median (IQR)	1 (0–2)	1 (0–3)	1 (0–2)		1 (0–2.5)	1 (0–3)	1 (0.2)	
PLT count (x10^9/L)	Mean (SD)	281.2 (214.9)	155.4 (105.6)	303.5 (221.7)	<0.0001	160.8 (119.9)	219.0 (146.3)	330.1 (239.8)	<0.0001
	Median (IQR)	221 (139–363)	122 (66–221)	250 (155–387)		123 (64–250)	171 (116–282)	278 (174–396)	
PLT count < 100 (x10^9/L)	*n* (%)	35 (12.8)	16 (39.0)	19 (8.2)	<0.0001	8 (38.1)	15 (17.1)	12 (7.3)	0.0001
Low‐intermediate 1 risk	*n* (%)	148 (55.9)	5 (12.8)	143 (63.3)	<0.0001	3 (15.8)	21 (24.1)	124 (78.0)	<0.0001
Intermediate 2‐ high risk	*n* (%)	117 (44.2)	34 (87.2)	83 (36.7)		16 (84.2)	66(75.9)	35 (22.0)	
Time to RUX start (years)	Mean (SD)	3.7 (4.7)	3.8 (3.6)	3.7 (4.8)	0.15	3.2 (2.9)	3.9 (4.4)	3.7 (5.0)	0.63
	Median (IQR)	2.1 (0.4–4.9)	3.2 (0.6–5.7)	1.9 (0.3–4.8)		2.4 (0.7–4.9)	2.6 (0.4–5.6)	1.9 (0.3–4.7)	
Duration of RUX (years)	Mean (SD)	2.6 (2.1)	1.5 (1.4)	2.8 (2.2)	0.0003	2.5 (2.1)	2.4 (2.2)	2.8 (2.1)	0.33
	Median (IQR)	2.2 (0.9–3.6)	1.1 (0.4–2.4)	2.4 (1.0–4.1)		1.8 (0.8–3.5)	2.0 (0.7–3.3)	2.3 (1.0–4.1)	
SCT	*n* (%)	32 (11.7)	5 (12.2)	27 (11.6)	0.99	2 (9.5)	12 (13.6)	18 (11.0)	0.78
Follow‐up (years)	Median (IQR)	2.5 (1.2–4.1)	2.0 (1.0–3.0)	2.6 (1.2–4.3)	0.02	2.5 (1.3–4.6)	2.5 (1.1–3.5)	2.5 (1.2–4.2)	0.77
Deaths	*n* (%)	108 (39.6)	26 (63.4)	82 (35.3)	0.0007	12 (57.1)	49 (55.7)	47 (28.7)	<0.0001
Time from RUX start to BP (years)	Median (IQR)	1.3 (0.7–2.2)	0.8 (0.4–1.5)	1.4 (0.9–2.4)	0.22	2.0 (2.0–2.0)	1.0 (0.5–2.0)	1.7 (1.1–2.4)	0.35

*Note*: Percentage calculated on variables'avialable data.

Abbreviations: BP, blast phase; CTCAE, common terminology criteria for adverse events; Hb, hemoglobin; IQR, interquartile range; MF, myelofibrosis; n, number; PLT, platelets; RBC‐TD, red blood cells‐transfusion dependency; RUX, ruxolitinib; SCT, stem cells transplant; SD, standard deviation; WBC, white blood cells count.

The period of diagnosis for C1 was between 1980 and 2021, and for C2 between 1989 and 2020—all locally updated [1]. Evolution to BP was defined by leukemic blast cells being at least 20% in peripheral blood or bone marrow [1, 3].

Anemia and its severity have been variously defined among different studies on MF; therefore we applied three prespecified distinctive categorizations. First, as for the common terminology criteria for adverse events (CTCAE) grading [21]: grade 3/4 anemia corresponded to Hb < 8 g/dL, grade 2 to Hb 8–10 g/dL, while grade 1 (Hb > 10 g/dL) anemia was grouped together with normal Hb values. Second, considering the sex‐ and severity‐adjusted Hb thresholds [22], we grouped patients into having severe (Hb < 8 g/dL in women and < 9 g/dL in men), moderate (Hb 8–9.9 g/dL in women and 9–10.9 g/dL in men), and mild/no anemia (Hb values higher than those defining moderate anemia). Lastly, we used the Hb 9.5 g/dL value as a threshold [23, 24]. Information on RBC transfusion dependence (RBC‐TD) was available only for the C2 dataset and defined as having received at least four RBC units in the previous 12 weeks [23, 24]. In C2, RBC‐TD status was dynamically evaluated, both at the time of RUX start, and after 6 months of treatment.

The study was approved by the Institutional Review Board of each Institution and conducted in accordance with the principles of the Declaration of Helsinki.

### 2.2 Statistical approach

Variables were summarized by conventional descriptive techniques: categorical variables by absolute and relative frequency; continuous by mean, standard deviation, median and interquartile range (IQR). Differences in baseline characteristics and follow‐up events were investigated among the pre‐specified anemia categories by applying chi‐square test for categorical variables and Wilcoxon or Kruskal Wallis test for continuous ones. International Prognostic Scoring System (IPSS), DIPSS, and MYSEC‐prognostic model (MYSEC‐PM) were properly applied to the two cohorts [5, 7, 12]. Follow‐up time was calculated as years between MF diagnosis (C1) or RUX start (C2) and the first event occurring among the following: JAKi start (only for C1), SCT, BP transformation, last contact date, death.

A Poisson regression model was applied to calculate BP incidence within 10 years of follow‐up, together with 95% confidence interval (95% CI) and hazard ratio (HR) in C1 and C2. Fatalities were evaluated regardless of BP evolution or SCT. A Kaplan–Meier curve was used to describe for C1 the probability of BP‐free survival, that is the time until BP evolution.

A log rank test was applied to compare survival times among the different Hb classes. By univariate Cox proportional hazards models, we evaluated associations between BP‐FS and Hb classes (C1 and C2) or RBC‐TD status (C2 only). The incidence rate ratio (IRR) with 95% CI was calculated to compare BP incidence rate between C1 and C2.

## 3 RESULTS

### 3.1 Patients’ characteristics

The main features at MF diagnosis and follow‐up events of the 1752 C1 subjects, overall and distinguished by anemia presence and degree, are detailed in Table 1 and in Table S2. Of those patients, 131 (7.5%) had CTCAE grade 3/4, 427 (24.4%) grade 2 and 1194 (68.1%) grade 1/no anemia. 208 (11.9%) had sex‐adjusted severe, 442 (25.2%) moderate and 1102 (62.9%) mild/no anemia. Hb was ≤9.5 g/dL in 419 (23.9%) cases. Anemia was more common and severe in overt‐ versus pre‐PMF and in PET‐ versus PPV‐MF (*p* < 0.0001). Considering all three pre‐specified anemia categorizations, older age, constitutional symptoms, higher circulating blasts, lower leukocytes, and platelet counts were associated with lower Hb in C1 cases (*p* < 0.0001). Five hundred seventy‐six (33.6%) patients were at intermediate‐2 or high‐risk, and they presented more frequently with anemia (*p* < 0.0001). JAKis were started in 285 (16.3%) of C1 patients: most (92.6%) received RUX as the first JAKi. Overall, SCT was performed in 87 (5%) of C1 cases, enriched in patients with lower Hb values at baseline (*all p* < 0.041). At a median follow‐up of 4 (IQR, 1.9–7.2) years, 890 (50.8%) patients died, and fatalities were reported mostly in patients presenting with a higher degree of anemia (*p* < 0.0001).

Table 2 and Table S3 summarize the characteristics of the 273 C2 patients at time of RUX start and subsequent events in the overall dataset, based on RBC‐TD status and on degree of anemia at the beginning of treatment. RBC‐TD was evident in 41 (15%) subjects. Twenty‐one patients (7.7%) showed CTCAE grade 3/4, 88 (32.2%) grade 2 and 164 (60.1%) grade 1/no anemia. 41 (15%) had sex‐adjusted severe, 91 (33.3%) moderate and 141 (51.7%) mild/no anemia. Hb was ≤9.5 g/dL in 79 (28.9%) cases. Of note, among RBC‐TD patients, 23 (56.1%) cases had CTCAE grade 2 anemia. Taking all three distinctive anemia classifications into account, in C2 dataset lower leukocytes (all *p* < 0.031) and platelet count (*p* < 0.0001) were more frequently detected in RBC‐TD cases and as Hb worsened. 117 (44.2%) C2 patients fell into the intermediate‐2 or high‐risk prognostic groups. Those latter more frequently included patients with baseline RBC‐TD or relevant anemia (*p* < 0.0001). RUX was started at a median time of 2.1 (IQR, 0.4–4.9) years after MF diagnosis. Median duration of RUX treatment was 2.2 (IQR, 0.9–3.6) years. SCT was performed in 32 (11.7%) subjects. At a median follow‐up time of 2.5 (IQR, 1.2–4.1) years, 108 (39.6%) patients died, and fatal events were more frequent in case of RBC‐TD and lower Hb values (all *p* < 0.00071).

### 3.2 BP incidence per anemia grade in the RUX‐unexposed population (C1)

Table 3 reports the rate and incidence of BP transformation in C1, distinguished by the presence and the degree of anemia at MF diagnosis. At a median time of 2.2 (IQR, 0.9–3.9) years from MF diagnosis (Table 1), BP evolution was reported in 185 (10.6%) C1 patients, with an incidence rate of 2.5% patients per year (p‐y) (95%CI, 2.2–2.9). The latter was higher in patients with greater degrees of anemia. Table S4 reports that, in univariate Cox model, HR for BP‐FS was 2.35 (95%CI, 1.72–3.20, *p* < 0.0001) in case of CTCAE grade 2 and 2.20 (95% CI, 1.32–3.68, *p* = 0.003) for grade 3/4 anemia, compared to patients with baseline grade1/no anemia. With respect to sex‐adjusted mild/no anemia, subjects with moderate and severe anemia showed a HR of 2.33 (95% CI, 1.69–3.22, *p* < 0.0001) and of 3.05 (95% CI, 2.03–4.57, *p* < 0.0001), respectively. Lastly, HR was 2.25 (95% CI, 1.66–3.06, *p* < 0.0001) if Hb was ≤9.5 g/dL, compared to higher Hb values.

**TABLE 3:** Blast phase prevalence and incidence of 1752 myelofibrosis patients (Cohort 1), in the overall dataset and based on anemia degree at diagnosis.

	*n* (%)	p‐y	incidence (% p‐y)	95% CI
Blast Phase	185 (10.6)	7337.67	2.5	2.2‐2.9
CTCAE Hb classification				
Grade 1/no anemia	102 (8.5)	5484.51	1.8	1.5–2.3
Grade 2 anemia	66 (15.5)	1458.31	4.5	2.7–7.5
Grade 3/4 anemia	17 (13.0)	394.86	4.3	2.1–8.7
Sex‐ and severity‐adjusted Hb classification				
Mild/ No anemia	88 (8.0)	5172.15	1.7	1.4–2.1
Moderate	64 (14.5)	1564.25	4.1	2.4–7.0
Severe	33 (15.9)	601.26	5.5	3.0–10.1
Hb 9.5 g/dL‐threshold classification				
> 9.5 g/dL	121 (9.1)	5991.14	2.0	1.7–2.4
≤9.5 g/dL	64 (15.3)	1346.53	4.8	2.9–7.7

Abbreviations: 95% CI, 95% confidence interval; CTCAE, common terminology criteria for adverse events; Hb, hemoglobin; n, number; p‐y, persons‐year.

**FIGURE 1:**
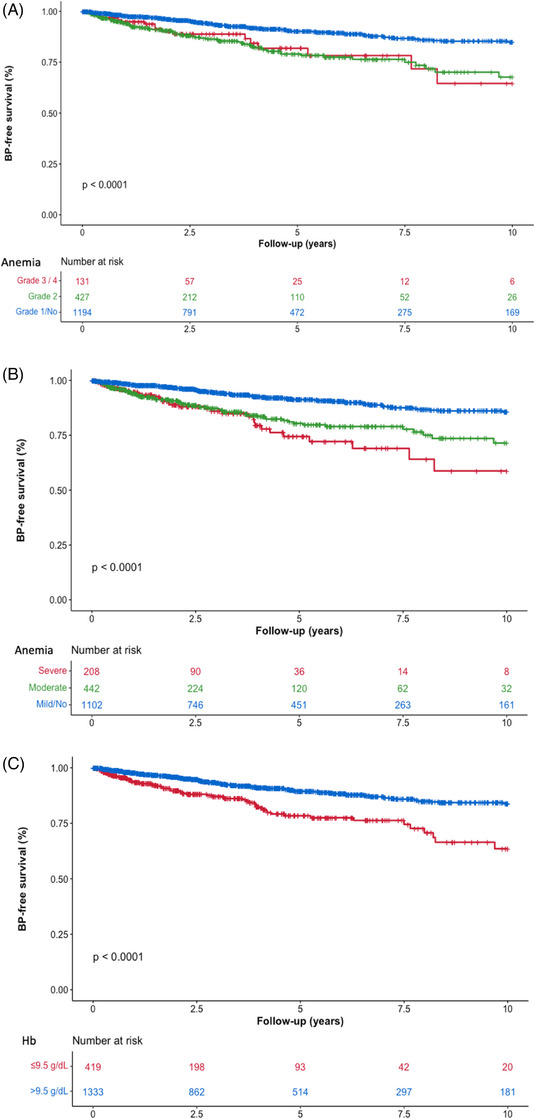
Association between blast phase‐free survival and anemia degree at time of myelofibrosis diagnosis in 1752 patients of Cohort 1. Kaplan–Meier curves describe the blast phase (BP)‐free survival of patients within the different anemia classes, as for common terminology criteria for adverse events (CTCAE, (A)), sex‐and severity‐adjusted (B) and as for hemoglobin (Hb) 9.5 g/dL‐threshold (C) categorization.

Figure 1 shows that BP‐FS was significantly associated (*p* < 0.0001) with the presence and severity of anemia at MF diagnosis, whichever of the three classifications is used (Figure 1A–C).

### 3.3 BP incidence per anemia grade and RBCs units’ requirement in patients on RUX (C2)

Table 4 summarizes prevalence and rates of BP transformation in C2 patients, based on RBC‐TD status and anemia degree at RUX start. At a median time of 1.3 (IQR, 0.7–2.2) years from RUX start (Table 2), BP evolution was documented in 23 (8.4%) C2 subjects with a BP incidence of 2.89% p‐y (95%CI, 1.91–4.35). Table S5 shows that, in univariate analysis, HR for BP‐FS was significantly higher in case of CTCAE grade 2 compared to grade1/no‐anemia (HR 2.36, 95% CI 1.03–5.40, *p* = 0.04), as it was for sex‐adjusted moderate with respect to mild/no‐anemia (HR 3.59, 95% CI 1.40–9.25, *p* = 0.01).

**TABLE 4:** Blast phase prevalence and incidence of 273 myelofibrosis patients treated with ruxolitinib (Cohort 2), in the overall dataset and based on red blood cells‐transfusion dependency and on anemia degree at treatment start.

	*n* (%)	p‐y	incidence (% p‐y)	95% CI
Blast Phase	23 (8.4)	796.1	2.89	1.91–4.35
RBC‐TD status				
no‐TD	19 (8.2)	709.73	2.68	0.34–20.97
TD	4 (9.8)	86.4	4.63	1.74–12.34
CTCAE Hb classification				
Grade 1/no anemia	10 (6.1)	487.1	2.05	1.10–3.82
Grade 2 anemia	12 (13.6)	246.9	4.86	1.1–20.91
Grade 3/4 anemia	1 (4.8)	62.1	1.61	0.11–23.38
Sex‐ and severity‐adjusted Hb classification				
Mild/No anemia	6 (4.2)	415.3	1.44	0.65–3.22
Moderate	14 (15.4)	271.0	5.16	0.89–29.92
Severe	3 (7.3)	109.7	2.73	0.30–24.33
Hb 9.5 g/dL‐threshold classification				
>9.5 g/dL	16 (8.2)	572.20	2.80	1.71–4.56
≤9.5 g/dL	7 (8.9)	233.89	3.13	0.80–12.40

Abbreviations: 95%CI, 95% confidence interval; CTCAE, common terminology criteria for adverse events; Hb, hemoglobin; n, number; p‐y, persons‐year; RBC‐TD, red blood cells‐transfusion dependency.

Considering the 41 RBC‐TD patients at time of RUX start, Table S6 reports that BP risk after six, 12, 18, and 24 months of treatment was higher compared to the 232 no‐TD subjects. Of note, in the RCB‐TD group, 75% of all BP transformations occurred within 1 year from RUX start. Within the no‐TD cohort, around 74% of evolutions were detected after 1 year of treatment.

Then, we analyzed 227 patients who received RUX for at least 6 months (median exposure 2.5 years) and available information on RBC units’ needs. At that time point, 47 (20.7%) were RBC‐TD. As reported in Table S7, BP was documented in 5 (10.1%) subjects of this subgroup, with an incidence rate of 4.2% p‐y (95%CI, 1.7–10.1). BP evolution occurred in 40% and 60% of cases within 12 and 24 months of treatment, respectively.

As detailed in Table 5, we found no difference in the IRR of BP between RUX‐unexposed C1 patients and RUX‐exposed C2 subjects, both when considering the whole populations and the different CTCAE anemia classes.

**TABLE 5:** Comparison of blast phase incidence between ruxolitinib‐unexposed Cohort 1 and Cohort 2 patients, in the overall datasets and based on anemia degree according to the CTCAE classification.

	C1		C2		Incidence rate ratio		
	Incidence (% p‐y)	95% CI	incidence (% p‐y)	95% CI	IRR	95% CI	*p*‐Value
Overall	2.5	2.2–2.9	2.89	1.9–4.4	0.87	0.56–1.41	0.53
CTCA grade 3/4 anemia	4.3	2.1–1.7	1.61	0.11–23.38	2.67	0.42–111.74	0.35
CTCA grade 2 anemia	4.5	2.7–7.5	4.86	1.1–20.91	0.93	0.49–1.89	0.80
CTCA grade 1/no anemia	1.8	1.5–2.3	2.05	1.1–3.82	0.91	0.47–1.95	0.74

Abbreviations: 95% CI, 95% confidence interval; C1, cohort 1; C2, cohort 2; CTCAE, common terminology criteria for adverse events; Hb, hemoglobin; IRR, incidence rate ratio; p‐y, persons‐year.

## 4 DISCUSSION

Anemia is a hallmark feature of PMF and SMF, being included not only within their diagnostic criteria but also among the major prognostic variables [1, 5, 7, 12, 22, 25].

An Italian study showed that 13% and 35% of pre‐ and overt‐PMF patients, respectively, presented Hb < 10 g/dL at diagnosis [4]. At SMF evolution, the MYSEC project reported that Hb was 11 g/dL as median and < 10 g/dL in 30% of cases [6, 20]. Lower Hb values were correlated with PET‐MF subtype, female gender, and grade 3 BMF [6, 26, 27].

In our large real‐world population of 1752 PMF and SMF subjects, median Hb value at diagnosis was 11.4 g/dL, and 31.9% of subjects presented CTCAE grade 2 or 3/4 anemia. Around 37% had sex‐adjusted moderate to severe anemia, while Hb was ≤9.5 g/dL in about 24% of cases. We confirmed a higher incidence of anemia in overt‐ versus pre‐PMF, and in PET‐ versus PPV‐MF patients.

In the DIPSS cohort, 47% of patients developed anemia after a median of 3.3 years from PMF diagnosis [12], and in the MYSEC population Hb values tended to decrease with a longer time to progression from PV [28]. Of 296 subjects included in the randomized phase 3 COMFORT studies, 134 (45.3%) had baseline Hb < 10 g/dL or required at least one RBC unit within 12 weeks prior to RUX initiation [29]. Similarly, in our real‐world cohort of 273 PMF and SMF cases, around 40% of subjects presented CTCAE grade 2 or 3/4 anemia at the time of RUX start.

In both our study populations, anemia was correlated with unfavorable features, like a cytopenic phenotype and the higher prognostic risk categories, as previously described [5, 7, 12, 22, 25, 30, 31]. We also confirmed that disease‐related RBC‐TD or anemia adversely impact on survival in RUX‐treated patients [16, 29].

Overall, around 10–20% of MF cases evolve into BP [4, 6, 8], with a consequent dismal survival [32, 33]. In an Italian study on 661 PMF cases, the cumulative 10‐years incidence of BP was 12% and 23% for pre‐ and overt‐PMF cases, respectively [4]. Within the MYSEC project, BP was reported in 20.5% patients after a median follow‐up of 3.5 years [20]. In a retrospective European cohort of 589 RUX‐treated PMF and SMF subjects, 14.5% progressed to BP after a median treatment time of 3 years, with a reported incidence of 3.7% p‐y [11].

In our 1752 PMF and SMF cases (33.6% at intermediate‐2 and high risk) largely unexposed to JAKis, 10.6% evolved into BP after a median time of 2.2 years from diagnosis, defining BP progression as an early event in the disease history (incidence: 2.5% p‐y). Among 273 prospectively observed PMF and SMF subjects (44.2% at intermediate‐2 and high risk) who started RUX after a median of 2.1 years from MF diagnosis, BP occurred in 8.4% of cases after a median time of 1.3 years from RUX start (incidence: 2.89% p‐y). No difference was found comparing rate of BP in our RUX‐unexposed versus ‐treated patients, also when stratified for CTCAE anemia classes.

Our results are aligned with previously reported rates of BP in the general MF population and in those receiving RUX [4, 11, 20], and they confirm that RUX leaves unaffected the probability of BP evolution in MF [11]. Difference in BP incidence between our RUX‐exposed cohort and the aforementioned European dataset could probably depend on population size and risk category distribution [11].

Risk factors for BP transformation have been studied mostly in PMF and before the widespread use of JAKis [9, 17, 18, 25, 34]. In this respect, Hb values < 10 g/dL are among the variables included in the DIPSS or in the molecularly annotated PMF prognostic models, that have been shown to predict BP transformation [17, 22, 25]. In over 1300 PMF subjects, the incidence of BP was higher in patients with sex‐adjusted moderate to severe anemia compared to lower grades [9]. Considering patients that had to receive at least one RBC unit to maintain Hb ≥8.5 g/dL at any time after PMF diagnosis, BP probability was significantly increased compared to subjects that did not receive RBC units [18]. Within 589 RUX‐treated MF patients, a greater risk of BP was associated with the higher DIPSS/MYSEC‐PM categories, while baseline RBC‐TD did not seem to play a role [11].

In our study, we confirmed an increased probability of BP progression in patients with relevant grades of anemia at PMF or SMF diagnosis. In RUX‐unexposed patients, BP incidence reached 4.3% and 4.5% p‐y for CTCAE grade 3/4 and grade 2 anemia, respectively. The same rate corresponded to 5.5% and 4.1% p‐y for sex‐adjusted severe and moderate anemia. Consequently, BP‐FS was significantly reduced in these subgroups compared to patients with milder anemia.

In the RUX‐exposed cohort, the trend was similar with an incidence of BP estimated to be 4.86% and 5.16% p‐y in CTCAE grade 2 anemia and sex‐adjusted moderate anemia, respectively. In patients with baseline RBC‐TD, the incidence of BP was 4.63% p‐y, slightly higher compared to non‐TD patients.

Based on these findings, treating anemia in MF represents a challenge, both due to its impacts on quality of life and its associated risk of BP progression, and avoiding the development of RBC‐TD remains a real unmet clinical need [2].

Conventional therapies for anemia, such as erythropoiesis‐stimulating agents and danazol allow some responses, but with very limited efficacy in RBC‐TD patients and short duration of effect [2, 17, 35, 36, 37, 38]. Hence, there is growing interest in finding innovative targets to improve anemia in MF. New molecules targeting the TGF‐beta superfamily, such as luspatercept and sotatercept, are under investigation alone or combined with RUX [23, 24, 39]. The former is already recommended by the National Comprehensive Cancer Network (NCCN) guidelines for MF patients with anemia [40]. KER050 is a modified ActRIIA ligand trap with effects on late and early erythropoiesis, and it is under evaluation in a phase 2 study [41]. Reduction of hepcidin overexpression is the target of the ALK inhibitor INCB000928 [42], and of the humanized monoclonal anti‐hemojuvelin DISC‐0974 [2]. Finally, ACVR1 inhibition has been found to be critical, with JAKis as momelotinib and pacritinib able to ameliorate Hb levels despite JAK inhibition [43, 44, 45, 46, 47]. It remains to be determined if these new molecules interfere with BP occurrence.

Our study reiterates the high frequency of anemia in a large cohort of MF patients, including more contemporary patients treated with JAKi, and assesses the impact of varying degrees of anemia on BP progression. Although conventional therapies for anemia have limited efficacy, new molecules under study appear to have the potential to raise Hb levels. Our study assessed the natural occurrence of post‐MF BP, that is left unaffected by RUX treatment, and these data will be a useful reference for physicians to make decisions on the efficacy and safety profile of innovative anemia treatments.

## AUTHOR CONTRIBUTIONS

BM and FP were involved in all stages of manuscript development, including drafting and critical reviewing of the manuscript. LB performed statistical analysis. All authors gave a substantial contribution to acquisition of data, revised critically the draft, and approved the submitted and final version of the paper.

## CONFLICT OF INTEREST STATEMENT

BM received honoraria for lecture from Novartis. FP received honoraria for lectures and advisory boards from Novartis, GSK, Bristol‐Myers Squibb/Celgene, Sierra Oncology, Abbvie, Janssen, Roche, AOP Orphan, Karyiopharma, Kyowa Kirin and MEI, Sumitomo. Other authors have no conflict of interest to disclose.

## ETHICS STATEMENT

The study was approved by the Institutional Review Board of Insubria (Varese, Italy) and conducted in accordance with the principles of the Declaration of Helsinki. The authors have confirmed patient consent statement is not applicable for this submission.

## Supporting information

Tables S1‐S7

## Data Availability

Data will be available for sharing upon request.
