# Supplementary material for: Incidence of blast phase in myelofibrosis according to anemia severity
Source: EJHaem. 2023 Jul 17;4(3):679–89. doi: 10.1002/jha2.745 (PMC10435699; doi:10.1002/jha2.745)
Supplement: Supplementary file 1 — Tables S1‐S7 [file JHA2-4-679-s001.docx]

Supplementary Information

Mora B et al.

**Incidence of blast phase in myelofibrosis according to anemia severity**

**Supplementary Table 1. Distribution of study population.**

|  | **Total** | **MPN-VA (C1)** | **DIPSS (C1)** | **MYSEC (C1)** | **RUXO-REL (C2)** |
| --- | --- | --- | --- | --- | --- |
| **Patients, n (%)** | 2381 (100) | 331 (13.9) | 519 (21.8) | 1258 (52.8) | 273 (11.5) |
| **Pre-PMF, n (%)** | 978 (41) | 157 (47.4) | - | - | 128 (46.9) |
| **Overt-PMF, n (%)** |  | 174 (52.6) | 519 (100.0) | - |  |
| **PET-MF, n (%)** | 690 (29) | - | - | 643 (51.1) | 47 (17.2) |
| **PPV-MF, n (%)** | 713 (30) | - | - | 615 (48.9) | 98 (35.9) |

**Legend:** MPN-VA= MPN database of the Varese Hematology Unit (Italy), C1= cohort 1, DIPSS= Dynamic International Prognostic Scoring System, MYSEC= Myelofibrosis Secondary to polycythemia vera and essential thrombocythemia, RUXO= ruxolitinib, REL= Rete Ematologica Lombarda, C2= cohort 2, n= number, pre-PMF= prefibrotic-primary myelofibrosis, overt-PMF= overt-primary myelofibrosis, PET-MF= post- essential thrombocythemia myelofibrosis, PPV-MF= post- polycythemia vera myelofibrosis.

**Supplementary Table 2. Main features at diagnosis and follow-up events of 1752 myelofibrosis patients in Cohort 1, stratified by anemia degree according to the sex- and severity-adjusted method and by the Hb 9.5 g/dL-threshold.**

|  |  | **Sex- and severity-adjusted Hb classification** | | | | **Hb 9.5 g/dL-threshold classification** | | |
| --- | --- | --- | --- | --- | --- | --- | --- | --- |
|  |  | **Severe** | **Moderate** | **Mild/no anemia** | **p-value** | **≤9.5 g/dL** | **>9.5 g/dL** | **p-value** |
| **Patients** | n (%) | 208 (11.9) | 442 (25.2) | 1102 (62.9) |  | 419 (23.9) | 1333 (76.1) |  |
| **Pre-PMF** | n (%) | 1 (0.5) | 7 (1.6) | 128 (11.6) | **<0.0001** | 5 (1.2) | 131 (9.8) | **<0.0001** |
| **Overt-PMF** | n (%) | 111 (53.4) | 171 (38.7) | 356 (32.3) |  | 190 (45.4) | 448 (33.6) |  |
| **PET-MF** | n (%) | 64 (30.8) | 168 (38.0) | 299 (27.1) |  | 151 (36.0) | 380 (23.5) |  |
| **PPV-MF** | n (%) | 32 (15.4) | 96 (21.7) | 319 (29.0) |  | 73 (17.4) | 374 (28.1) |  |
| **Age at diagnosis (years)** | mean (SD) | 65.9 (10.5) | 65.3 (11.6) | 60.6 (13.3) | **<0.0001** | 65.7 (11.4) | 61.4 (13.0) | **<0.0001** |
|  | median (IQR) | 67 (59-73) | 66 (58-73) | 62 (52-71) |  | 67 (58-73) | 63 (53-71) |  |
| **Male gender** | n (%) | 175 (84.1) | 248 (56.1) | 564 (57.2) | **<0.0001** | 245 (58.5) | 742 (55.7) | 0.31 |
| **Palpable spleen** | n (%) | 158 (79.0) | 331 (80.2) | 803 (75.5) | 0.13 | 316 (79.8) | 976 (76.3) | 0.14 |
| **Palpable spleen (cm)** | mean (SD) | 8.1 (5.6) | 7.5 (5.4) | 7.9 (5.4) | 0.32 | 7.8 (5.6) | 7.8 (5.4) | 0.74 |
|  | median (IQR) | 7 (4-11) | 6 (3-10) | 7 (4-11) |  | 6 (3-11) | 7 (4-11) |  |
| **Constitutional symptoms** | n (%) | 105 (50.5) | 156 (35.3) | 271 (24.6) | **<0.0001** | 186 (44.4) | 346 (26.0) | **<0.0001** |
| **Hb (g/dL)** | mean (SD) | 7.5 (1.1) | 9.6 (0.7) | 12.8 (1.6) | **<0.0001** | 8.2 (1.1) | 12.3 (1.8) | **<0.0001** |
|  | median (IQR) | 7.7 (6.9-8.3) | 9.6 (9.1-10.0) | 12.6 (11.6-13.9) |  | 8.5 (7.7-9.0) | 12.1 (10.9-13.6) |  |
| **Circulating blasts (%)** | mean (SD) | 1.3 (2.5) | 1.1 (2.6) | 0.6 (1.8) | **<0.0001** | 1.2 (2.7) | 0.6 (1.9) | **<0.0001** |
|  | median (IQR) | 0 (0-2) | 0 (0-1) | 0 (0-0) |  | 0 (0-1) | 0 (0-0) |  |
| **WBC (x10^9/L)** | mean (SD) | 10.2 (11.4) | 12.3 (12.4) | 13.8 (10.7) | **<0.0001** | 11.8 (12.8) | 13.4 (10.8) | **<0.0001** |
|  | median (IQR) | 6.6 (3.7-11.3) | 8.0 (5.3-14.3) | 10.9 (7.3-16.6) |  | 7.6 (4.5-13.6) | 10.3 (7.0 -16.0) |  |
| **PLT count (x10^9/L)** | mean (SD) | 274.5 (266.5) | 344.8 (274.3) | 469.5 (327.9) | **<0.0001** | 304.6 (273.6) | 449.7 (321.7) | **<0.0001** |
|  | median (IQR) | 198.5 (82.5-363.0) | 256 (146-485) | 402.5 (238-609) |  | 219 (112-418.5) | 390.5 (211.5-600) |  |
| **PLT count <100 (x10^9/L)** | n (%) | 60 (28.9) | 70 (16.1) | 60 (5.5) | **<0.0001** | 90 (21.6) | 100 (7.5) | **<0.0001** |
|  |  |  |  |  |  |  |  |  |
| **Low-intermediate 1 risk** | n (%) | 41 (20.4) | 173 (40.6) | 926 (85.0) | **<0.0001** | 102 (25.2) | 1038 (79.2) | **<0.0001** |
| **Intermediate 2- high risk** | n (%) | 160 (79.6) | 253 (59.4) | 163 (15.0) |  | 303 (74.8) | 273 (20.8) |  |
| **JAKi exposure during time** | n (%) | 24 (11.5) | 64 (14.5) | 197 (17.9) | **0.04** | 57 (13.6) | 228 (17.1) | 0.09 |
| **Time to JAKi start (years)** | mean (SD) | 2.3 (1.7) | 3.5 (3.7) | 4.2 (3.9) | **0.01** | 3.1 (2.9) | 4.0 (3.9) | 0.08 |
|  | median (IQR) | 1.5 (1.1-3.0) | 1.9 (0.8-4.4) | 3.1 (1.4-5.6) |  | 2.1 (1.1-4.3) | 2.9 (1.2-5.3) |  |
| **SCT** | n (%) | 17 (8.2) | 26 (5.9) | 44 (4.0) | **0.02** | 30 (7.2) | 57 (4.3) | **0.02** |
| **Follow-up (year)** | median (IQR) | 2.3 (1.0-4.4) | 3.1 (1.2-5.8) | 4.8 (2.3-8.5) | **<0.0001** | 2.8 (1.1-5.2) | 4.5 (2.1-8.0) | **<0.0001** |
| **Deaths** | n (%) | 165 (79.3) | 265 (60.0) | 460 (41.7) | **<0.0001** | 294 (70.2) | 596 (44.7) | **<0.0001** |
| **Time to BP (years)** | median (IQR) | 1.8 (0.8-3.9) | 1.5 (0.7-3.4) | 2.6 (1.1-4.7) | 0.16 | 1.7 (0.6-3.9) | 2.5 (1.0-4.2) | 0.16 |

***Percentage calculated on variables’avialable data.***

**Legend:** n= number, pre-PMF= prefibrotic-primary myelofibrosis, overt-PMF= overt-primary myelofibrosis, PET-MF= post- essential thrombocythemia myelofibrosis, PPV-MF= post- polycythemia vera myelofibrosis, SD= standard deviation, IQR= interquartile range, WBC= white blood cells count, PLT= platelets, JAKi= JAK inhibitors, MF= myelofibrosis, SCT= stem cells transplant, BP= blast phase.

**Supplementary Table 3. Main features at ruxolitinib start and follow-up events of 273 patients in Cohort 2, stratified by anemia degree according to the sex- and severity-adjusted method and by the Hb 9.5 g/dL-threshold.**

|  |  | **Sex- and severity-adjusted Hb classification** | | | | | **Hb 9.5 g/dL-threshold classification** | | | |
| --- | --- | --- | --- | --- | --- | --- | --- | --- | --- | --- |
|  |  | **Severe** | **Moderate** | **Mild/no anemia** | **p-value** | **≤9.5 g/dL** | | **>9.5 g/dL** | **p-value** |  |
| **Patients** | n (%) | 41 (15) | 91 (33.3) | 141 (51.7) |  | 79 (28.9) | | 194 (71.1) |  |  |
| **Age at diagnosis (years)** | mean (SD) | 64.1 (9.7) | 61.6 (11.5) | 59.4 (13.0) | 0.05 | 62.1 (11.1) | | 60.4 (12.5) | 0.19 |  |
|  | median (IQR) | 66 (59-71) | 63 (56-69) | 61 (53-68) |  | 64 (57-69) | | 61 (54-69) |  |  |
| **Male gender** | n (%) | 35 (85.4) | 63 (69.2) | 70 (49.7) | **<0.0001** | 55 (69.6) | | 113 (58.3) | 0.08 |  |
| **Palpable spleen** | n (%) | 39 (100.0) | 88 (98.9) | 132 (96.4) | 0.44 | 75 (98.7) | | 184 (97.4) | 0.68 |  |
| **Palpable spleen (cm)** | mean (SD) | 10.5 (5.3) | 12.3 (6.0) | 10.7 (5.3) | 0.13 | 11.5 (6.0) | | 11.1 (5.4) | 0.95 |  |
|  | median (IQR) | 9 (6-14) | 12 (8-15) | 10.5 (7-14) |  | 10 (7-15) | | 11 (8-14.7) |  |  |
| **Constitutional symptoms** | n(%) | 24 (58.5) | 54 (59.3) | 99 (70.2) | 0.16 | 51 (64.6) | | 126 (65.0) | 0.95 |  |
| **Hb (g/dL)** | mean (SD) | 7.8 (0.8) | 9.6 (0.6) | 12.3 (1.3) | **<0.0001** | 8.4 (0.9) | | 11.7 (1.5) | **<0.0001** |  |
|  | median (IQR) | 7.8 (8.5-7.2) | 9.7 (9.2-10.1) | 12.2 (11.2-13.3) |  | 8.7 (7.7-9.1) | | 11.4 (10.3-13.0) |  |  |
| **Hb >10 (g/dL)** | n (%) |  |  |  |  |  | |  |  |  |
| **Hb 8-10 (g/dL)** | n (%) |  |  |  |  |  | |  |  |  |
| **Hb <8 (g/dL)** | n (%) |  |  |  |  |  | |  |  |  |
|  |  |  |  |  |  |  | |  |  |  |
| **Mild/no anemia** | n (%) |  |  |  |  |  | |  |  |  |
| **Moderate anemia** | n (%) |  |  |  |  |  | |  |  |  |
| **Severe anemia** | n (%) |  |  |  |  |  | |  |  |  |
|  |  |  |  |  |  |  | |  |  |  |
| **Hb ≤9.5 (g/dL)** | n (%) |  |  |  |  |  | |  |  |  |
| **WBC (x10^9/L)** | mean (SD) | 10.3 (8.3) | 16.2 (14.5) | 14.2 (9.2) | **0.009** | 12.8 (11.8) | | 14.9 (11.0) | **0.01** |  |
|  | median (IQR) | 7.2 (4.9-13.6) | 10.3 (6.4-20.6) | 11.7 (7.7-16.6) |  | 9.26 (5.31-16.20) | | 11.33 (7.30-18.69) |  |  |
| **Circulating blasts (%)** | mean (SD) | 1.8 (2.7) | 1.9 (2.4) | 1.2 (1.9) | **0.03** | 2.0 (2.7) | | 1.4 (2.0) | 0.10 |  |
|  | median (IQR) | 1 (0-2) | 1 (0-3) | 0.9 (0-1.7) |  | 1 (0-2.5) | | 1 (0-2) |  |  |
| **PLT count (x10^9/L)** | mean (SD) | 157.8 (110.3) | 266.9 (205.5) | 326.4 (229.0) | **<0.0001** | 184.5 (141.4) | | 320.6 (227.2) | **<0.0001** |  |
|  | median (IQR) | 122 (66-201) | 212 (137-359) | 274 (176-397) |  | 153 (92-220) | | 272 (171-396) |  |  |
| **PLT count <100 (x10^9/L)** | n (%) | 15 (36.6) | 9 (9.9) | 11 (7.8) | **<0.0001** | 21 (26.6) | | 14 (7.2) | **<0.0001** |  |
| **Low-intermediate 1 risk** | n (%) | 7 (18.0) | 26 (29.6) | 115 (83.3) | **<0.0001** | 12 (15.8) | | 136 (72.0) | **<0.0001** |  |
| **Intermediate 2- high risk** | n (%) | 32 (82.0) | 62 (70.4) | 23 (16.7) |  | 64 (84.2) | | 53 (28.0) |  |  |
| **Time to RUX start (years)** | mean (SD) | 2.8 (3.2) | 3.8 (4.3) | 3.9 (5.2) | 0.51 | 3.1 (3.6) | | 4.0 (5.0) | 0.45 |  |
|  | median (IQR) | 1.8 (0.3-4.1) | 2.4 (0.6-5.3) | 1.9 (0.3-5.0) |  | 1.9 (0.3-4.2) | | 2.4 (0.4-5.0) |  |  |
| **Duration of RUX (years)** | mean (SD) | 2.2 (1.9) | 2.6 (2.2) | 2.8 (2.1) | 0.27 | 2.5 (2.0) | | 2.7 (2.2) | 0.42 |  |
|  | median (IQR) | 1.8 (0.7-3.3) | 2.1 (0.8-4.1) | 2.4 (1.1-3.9) |  | 2.1 (0.8-3.4) | | 2.3 (0.9-3.9) |  |  |
| **SCT** | n (%) | 6 (14.6) | 9 (9.9) | 17 (12.1) | 0.72 | 10 (12.7) | | 22 (11.3) | 0.76 |  |
| **Follow-up (years)** | median (IQR) | 2.5 (1.2-3.4) | 2.5 (1.1-4.2) | 2.5 (1.2-4.2) | 0.93 | 2.5 (1.3-3.5) | | 2.5 (1.1-4.2) | 0.93 |  |
| **Deaths** | n (%) | 24 (58.5) | 45 (49.5) | 39 (27.7) | **0.0001** | 44 (55.7) | | 64 (33.0) | **0.0005** |  |
| **Time from RUX start to BP (years)** | median (IQR) | 1.9 (0.7-2.0) | 1.1 (0.2-2.5) | 1.7 (1.2-2.2) | 0.81 | 1.3 (0.7-2.0) | | 1.3 (0.6-2.3) | 0.87 |  |

***Percentage calculated on variables’avialable data.***

**Legend:** RBC-TD= red blood cells-transfusion dependency, Hb= hemoglobin, n= number, SD= standard deviation, IQR= interquartile range, WBC= white blood cells count, PLT= platelets, MF= myelofibrosis, RUX= ruxolitinib, SCT= stem cells transplant, BP= blast phase.

**Supplementary Table 4. Results of the univariate analysis to identify a correlation between anemia degree and blast phase-free survival in 1752 myelofibrosis patients of Cohort 1.**

|  |  | | |
| --- | --- | --- | --- |
|  | **HR** | **95%CI** | **p-value** |
| **CTCAE Hb classification** |  |  |  |
| Grade 1/no anemia | 1.0 | Reference |  |
| Grade 2 anemia | 2.35 | 1.72-3.20 | **<0.0001** |
| Grade 3/4 anemia | 2.20 | 1.32-3.68 | **0.003** |
|  |  |  |  |
| **Sex- and severity-adjusted Hb classification** |  |  |  |
| Mild/no anemia | 1.0 | Reference |  |
| Moderate anemia | 2.33 | 1.69-3.22 | **<0.0001** |
| Severe anemia | 3.05 | 2.03-4.57 | **<0.0001** |
|  |  |  |  |
| **ACE-536-MF-001 Hb classification** |  |  |  |
| Hb ≤9.5 g/dL | 1.0 | Reference |  |
| Hb >9.5 g/dL | 2.25 | 1.66-3.06 | **<0.0001** |

**Legend**: HR= hazard ratio, 95%CI= 95% confidence interval, CTCAE= Common Terminology Criteria for Adverse Events, Hb= hemoglobin.

**Supplementary Table 5. Results of the univariate analysis to identify a correlation between red blood cells-transfusion dependency or anemia degree and blast phase-free survival in 273 myelofibrosis patients at time of ruxolitinib start (Cohort 2).**

|  | **HR** | **95%CI** | **p-value** |
| --- | --- | --- | --- |
| **RBC-TD status** |  |  |  |
| no -TD | 1.0 | Reference |  |
| TD | 1.48 | 0.50-4.35 | 0.48 |
| **CTCAE Hb classification** |  |  |  |
| Grade 1/no anemia | 1.0 | Reference |  |
| Grade 2 anemia | 2.36 | 1.03-5.40 | **0.04** |
| Grade 3/4 anemia | 0.79 | 0.10-6.06 | 0.81 |
| **Sex- and severity-adjusted Hb classification** |  |  |  |
| Mild/ No anemia | 1.0 | Reference |  |
| Moderate | 3.59 | 1.40-9.25 | **0.01** |
| Severe | 1.79 | 0.45-7.13 | 0.41 |
| **Hb 9.5 g/dL-threshold classification** |  |  |  |
| >9.5 g/dL | 1.0 | Reference |  |
| ≤9.5 g/dL | 1.06 | 0.44-2.56 | 0.90 |

**Legend**: HR= hazard ratio, 95%CI= 95% confidence interval, CTCAE= Common Terminology Criteria for Adverse Events, Hb= hemoglobin.

**Supplementary Table 6. Blast phase prevalence and incidence at different timepoints in 273 myelofibrosis patients treated with ruxolitinib (Cohort 2), based on red blood cells-transfusion dependency at treatment start.**

| **Blast Phase** | **n (%)** | **p-y** | **incidence (% p-y)** | **95%CI** |
| --- | --- | --- | --- | --- |
| **at 6 months** |  |  |  |  |
| no-TD | 4 (1.7) | 114.85 | 3.48 | 0.05-221.20 |
| TD | 1 (2.4) | 20.16 | 4.96 | 0.70-35.2 |
| **at 12 months** |  |  |  |  |
| no-TD | 5 (2.1) | 228.77 | 2.19 | 0.17-28.36 |
| TD | 3 (7.3) | 39.75 | 7.54 | 2.43-23.40 |
| **at 18 months** |  |  |  |  |
| no-TD | 10 (4.3) | 340.91 | 2.93 | 0.26-33.04 |
| TD | 3 (7.3) | 58.75 | 5.11 | 1.64-15.83 |
| **at 24 months** |  |  |  |  |
| no-TD | 12 (5.2) | 451.89 | 2.66 | 0.24-29.17 |
| TD | 3 (7.3) | 77.75 | 3.86 | 1.24-12.0 |

**Legend:** n= number, p-y= persons-year, 95%CI= 95% confidence interval, TD= red blood cells-transfusion dependency.

**Supplementary Table 7. Blast phase prevalence and incidence at different timepoints in 47 myelofibrosis patients with red blood cells-transfusion dependency after six months of ruxolitinib (Cohort 2).**

| **Blast Phase** | **n (%)** | **p-y** | **incidence (% p-y)** | **95%CI** |
| --- | --- | --- | --- | --- |
| **Total** | 5 (10.1) | 118.88 | 4.2 | 1.7-10.1 |
| **At 12 months** | 2 (4.2) | 46.83 | 4.3 | 1.1-17.1 |
| **At 18 months** | 2 (4.2) | 69.33 | 2.9 | 0.7-11.5 |
| **At 24 months** | 3 (6.4) | 91.82 | 3.3 | 1.1-10.1 |

**Legend:** n= number, p-y= persons-year, 95%CI= 95% confidence interval.
